# A Novel C1q Domain-Containing Protein Isolated from the Mollusk *Modiolus kurilensis* Recognizing Glycans Enriched with Acidic Galactans and Mannans

**DOI:** 10.3390/md19120668

**Published:** 2021-11-26

**Authors:** Andrei V. Grinchenko, Alex von Kriegsheim, Nikita A. Shved, Anna E. Egorova, Diana V. Ilyaskina, Tatiana D. Karp, Nikolay V. Goncharov, Irina Y. Petrova, Vadim V. Kumeiko

**Affiliations:** 1 A.V. Zhirmunsky National Scientific Center of Marine Biology, Far Eastern Branch, Russian Academy of Sciences, 690041 Vladivostok, Russia; grishagrin@mail.ru (A.V.G.); nikitawayfarer@yandex.ru (N.A.S.); goncharovnv.GN@gmail.com (N.V.G.); iupet@mail.ru (I.Y.P.); 2 Institute of Genetics and Cancer, The University of Edinburgh, Edinburgh EH4 2XU, UK; Alex.VonKriegsheim@ed.ac.uk; 3 Institute of Life Sciences and Biomedicine, Far Eastern Federal University, 690922 Vladivostok, Russia; bioanna1995@gmail.com (A.E.E.); Ilyaskinadiana0506@gmail.com (D.V.I.); tachellabio@gmail.com (T.D.K.)

**Keywords:** bivalve mollusk, C1q domain-containing, lectin-like, pattern recognition receptor, polysaccharides, interstitial compartment

## Abstract

C1q domain-containing (C1qDC) proteins are a group of biopolymers involved in immune response as pattern recognition receptors (PRRs) in a lectin-like manner. A new protein MkC1qDC from the hemolymph plasma of *Modiolus kurilensis* bivalve mollusk widespread in the Northwest Pacific was purified. The isolation procedure included ammonium sulfate precipitation followed by affinity chromatography on pectin-Sepharose. The full-length MkC1qDC sequence was assembled using de novo mass-spectrometry peptide sequencing complemented with N-terminal Edman’s degradation, and included 176 amino acid residues with molecular mass of 19 kDa displaying high homology to bivalve C1qDC proteins. MkC1qDC demonstrated antibacterial properties against Gram-negative and Gram-positive strains. MkC1qDC binds to a number of saccharides in Ca^2+^-dependent manner which characterized by structural meta-similarity in acidic group enrichment of galactose and mannose derivatives incorporated in diversified molecular species of glycans. Alginate, κ-carrageenan, fucoidan, and pectin were found to be highly effective inhibitors of MkC1qDC activity. Yeast mannan, lipopolysaccharide (LPS), peptidoglycan (PGN) and mucin showed an inhibitory effect at concentrations three orders of magnitude greater than for the most effective saccharides. MkC1qDC localized to the mussel hemal system and interstitial compartment. Intriguingly, MkC1qDC was found to suppress proliferation of human adenocarcinoma HeLa cells in a dose-dependent manner, indicating to the biomedical potential of MkC1qDC protein.

## 1. Introduction

The specific interaction of proteins with carbohydrate underlies a variety of cell-to-cell communications and cooperation with extracellular matrix (ECM). Cell surface forms unique glycosylation patterns, which play a significant role in the immune defense providing the self and non-self-discrimination abilities. Meanwhile, the most known group of carbohydrate binding proteins are lectins, which demonstrated diversified affinity to a variety of carbohydrate determinants. Initially all carbohydrate binding proteins were recognized as lectins. However, extensive genomic research distinguished separate groups of proteins recognizing glycans of pathogen-associated molecular patterns (PAMPs) [1]. Among these biopolymers, the C1qDC proteins form very interesting family widespread in different groups of invertebrates that probably predicts an origin of vertebrates complement system involved in the immune response. 

In the vertebrates, the C1q protein is a key component of the complement system, triggering the classical pathway of its activation. In invertebrates, C1q domains and corresponding proteins show a wide range of ligands, including a variety of PAMPs. Interestingly, the largest number of genes encoding C1qDC proteins was found in bivalve species genomes: 337 in *Crassostrea gigas* [2], 296 in *Pinctada fucata* [3], 445 in *Modiolus philippinarum* [4], 1182 in *Ruditapes philippinarum* [5], 554 in *Saccostrea glomerata* [6] and 476 in *Crassostrea virginica* [7]. This unusually large number of genes probably arose due to multiple duplications of genomic fragments. Studies suggest that this process occurred independently in different species [5,7]. Presumably, the abundance of C1qDC proteins allows covering the protective needs of the bivalve against various pathogens due to the potential structural diversity of PAMPs. Many bivalve C1qDC proteins are soluble, secreting PRRs that agglutinate and opsonize foreign agents by PAMPs recognizing [8,9,10,11,12], but several studies also have shown that they are involved in embryogenesis [9,10,13], shell formation [14,15,16] and interaction with predators [14]. Until recent years, some bivalve C1qDC proteins are classified either as lectins [8,17,18,19] or as lectin-like proteins, which emphasizes their probable origin as lectins with subsequent diversification [2,7,20].

Currently, lectins as biomedical and biotechnological tools are a very active area of research [21,22,23,24,25]. In recent years, great attention has been paid to carbohydrate binding proteins derived from marine organisms. Bivalves have the most extensive repertoires of lectins, which allows them to thrive in an environment saturated with pathogens [26]. In addition to antimicrobial properties, bivalve lectins show promising antitumor and antiviral activity [22,24]. Bivalve C1qCD proteins are a large group of carbohydrate-recognizing molecules which are interesting due to functional and property similarities with lectins and can be a new object of biotechnological usage.

In this work, a novel C1qDC bivalve protein from *M. kurilensis* (MkC1qDC) was identified and an effective protocol for its isolation was developed. Immunohistochemical detection showed intracellular localization of target protein only in hemocytes and MkC1qDC presence to the hemal system, ECM and interstitial components. The physicochemical and functional properties of MkC1qDC was characterized, including the carbohydrate specificity and antimicrobial activity. Furthermore, MkC1qDC demonstrated the inhibition of HeLa proliferation in a dose-dependent manner, suggesting biomedical potential of this protein.

## 2. Results

### 2.1. MkC1qDC Purification and Electrophoretic Properties

**Figure 1:**
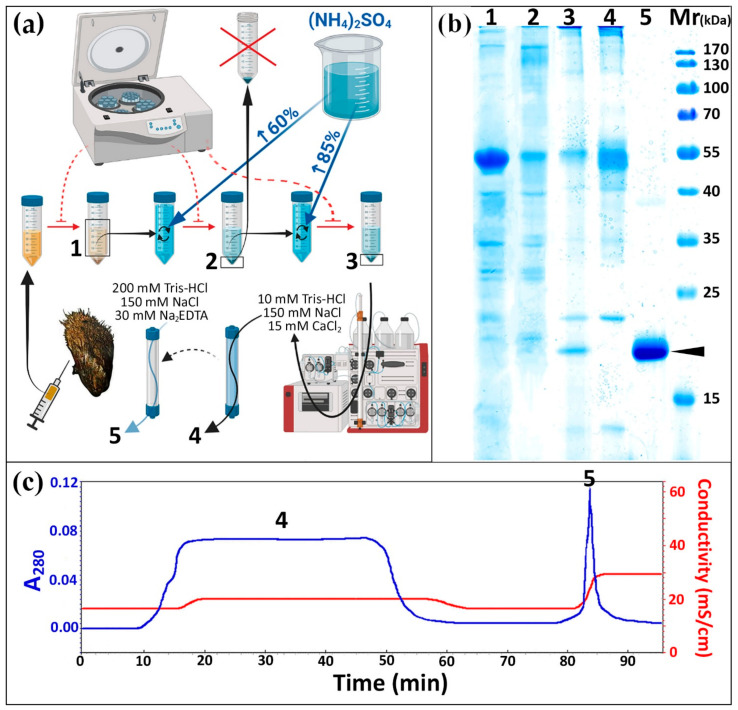
The scheme of MkC1qDC isolation (**a**); sodium dodecyl sulfate polyacrylamide gel electrophoresis (SDS-PAGE) of samples obtained at different stages of MkC1qDC isolation (**b**); MkC1qDC band labeled; elution profile of affinity chromatography on pectin-Sepharose CL-4B (**c**). 1—cell-free hemolymph; 2—sample after precipitation by ammonium sulfate 0–60% saturation; 3—second precipitation by ammonium sulfate before affinity chromatography; 4—flow-through of affinity chromatography; 5—eluted fraction; Mr—molecular weight standards.

Cell-free hemolymph (plasma) of *M. kurilensis* is characterized by the highest hemagglutination (HA) activity towards human erythrocytes equally for all groups of the AB0 system displaying 1/64–1/256 titers against 6 × 10^7^ cells per mL. Analysis of the carbohydrate specificity of *M. kurilensis* plasma agglutinins by hemagglutination inhibition (HAI) assay showed that mucins type II and type III, mannan, N-acetyl-D-galactosamine, N-acetyl-D-glucosamine, sialic acid, D-(–)-ribose, as well as D-glucuronic and D-galacturonic acids, were inhibitory. Fraction 0–15% of ammonium sulfate precipitation of plasma proteins had no agglutinating activity. Fractions 15−30%, 30−45%, 45−60%, 60−75% and 75−85% had agglutinating activity, but only 60−75% and 75−85% was inhibited by uronic acids and citrus pectin containing a polygalacturonic acid structure. The inhibition activity was Ca^2+^-dependent as 30 mM Na_2_EDTA disposed of it. Thus, in the presented final scheme for the isolation of the target protein (Figure 1a), ammonium sulfate precipitation was reduced to two fractions: 0–60% and 60–85%. Many of the major plasma proteins (Figure 1b, Lane 1), such as 41 kDa, 36 kDa, 29 kDa and some others, was found only in the 0–60% fraction (Figure 1b, Lane 2), while in the fraction 60–85% there were significantly fewer bands, but as a result of concentration, a band with a mass of 19 kDa, corresponding to the target MkC1qDC protein, appears (Figure 1b, Lane 3). After chromatography purification of the 60−85% fraction using pectin-immobilized affinity column (Figure 1c) the band disappeared in the flow-through (Figure 1b, Lane 4), but was recovered in the Ca^2+^-binding eluate (Figure 1b, Lane 5). Typically, the protein eluted as a narrow peak upon changing the eluate solution, as noticed by an increase in conductivity (Figure 1c). On average, we were able to purify 2-4 mg of MkC1qDC from 1000 mL of *M. kurilensis* hemolymph plasma.

### 2.2. Sequencing Analysis

**Figure 2:**
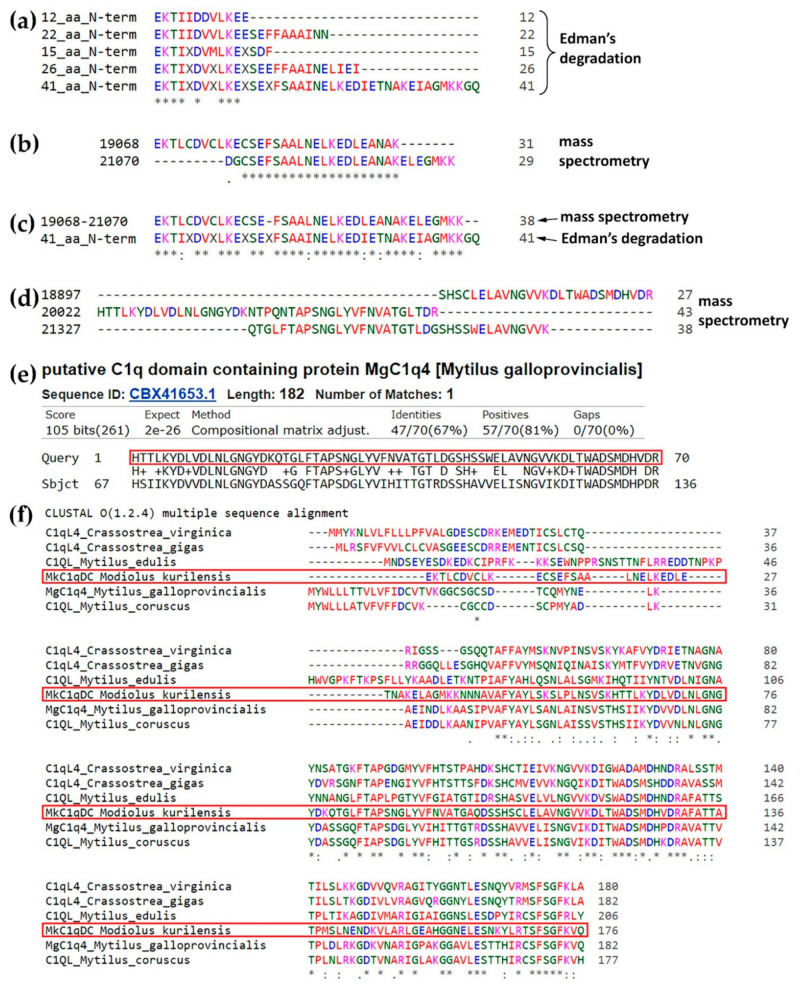
Amino acids sequencing of MkC1qDC: N-terminal peptides from Edman’s degradation (**a**); alignment of two fragments obtained by mass-spectrometric de novo sequencing (**b**) identical to Edman’s degradation results (**c**); alignment of three de novo peptides (**d**) that had high quality of matches with C1qDC or C1q-like proteins from other Bivalvia (**e**); alignment of full-length MkC1qDC sequence assembly with nearest homologues (**f**).

We initially sequenced the protein by Edman sequencing. MkC1qDC samples were purified independently at five different time points, and we were able to sequence 13 to 41 residues (Figure 2a). However, we were unable to find any significant matches against any of the public databases when using the Basic Local Alignment Search Tool (BLAST, https://blast.ncbi.nlm.nih.gov/Blast.cgi, accessed on 26 May 2021).

To complement the data, we applied de novo mass spectrometry peptide sequencing to determine the amino acid sequence of the protein. The purified protein was digested with trypsin and several peptides of up to 50 residues with an Average Local Confidence >80% were identified. Analysis of these fragments allowed us to assemble a sequence that matched to data obtained by Edman sequencing (Figure 2b,c). In addition, a 70 amino acids sequence was generated by alignment of three overlapping peptides (Figure 2d). Analysis of this fragment by BLAST found a match with C1qDC or C1q-like proteins of Bivalvia with high statistical significance and up to 67% identity for CBX41653.1 (Figure 2e and Figure S1). The full-length MkC1qDC sequence (Figure S2) was subsequently assembled using the longer stretches obtained by using the de Bruijn assembler ALPS [27] and CBX41653.1 as a reference. Analysis of assembled MkC1qDC sequence by BLAST detected a putative conserved C1q superfamily domain and high homology to other Bivalvia C1qDC proteins: CBX41653.1; CAC5364865.1; CAG2251157.1; XP_022294274.1; XP_034307311.1 (Figure 2f). The homologous proteins had one C1q domain at the C-terminus, a short unique N-terminus and were in the range of 177−206 residues. Full-length MkC1qDC has 176 amino acids in length, a predicted molecular weight of 19181 Da and a pI 5.2.

### 2.3. Physicochemical Properties

**Figure 3:**
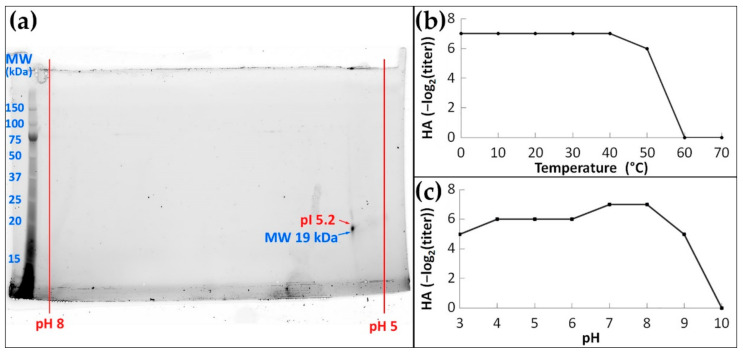
Basic physicochemical properties of the MkC1qDC: 2D electrophoresis of purified MkC1qDC (**a**); the activity after 1 h of incubation at different temperatures (**b**) or pH (**c**). HA: hemagglutination.

To further explore the properties of MkC1qDC we used 2D electrophoresis. Purified MkC1qDC was found to be a single polypeptide with a molecular mass of 19 kDa mass (Figure 3a) and a pI of 5.2, both of which match the theoretical pI and molecular weight.

Purified MkC1qDC had high agglutinating activity after incubation for 1 h at temperatures of 0–40 °C, which slightly decreased at 50 °C, and were completely lost at 60 °C or higher (Figure 3b). At the same time, the maximum HA activity was detected after 1 h of incubation in the pH range of 7–8 (Figure 3c). Increasing the pH to nine led to a slight decrease in activity and no activity was detected above pH 10. Acidic solutions up to pH 3 had only a weak effect on HA activity, indicating the wide range of pH stability.

### 2.4. Carbohydrate Specificity

The highest MkC1qDC agglutination activity was against human erythrocytes with no prevalence with respect to blood group. Therefore, all succeeding experiments was performed only with human group 0 erythrocytes.

Among 23 monosaccharides tested by the HAI assay, only six were inhibitory (Table 1). L-gulose, sialic (N-acetylneuraminic), D-galacturonic and D-glucuronic acids showed the highest inhibitory effect among monosaccharides with half maximal inhibitory concentration (IC50) in the range of 1.16–1.46 mg/mL. D-galactose and 2-deoxy-D-galactose were effective in the range of 2.46–5.4 mg/mL. Furthermore, disaccharides such as 2α-mannobiose and D-lactose were noted as inhibitory molecules displaying IC50s starting from 5.13 mg/mL. Mannan from *Saccharomyces cerevisiae*, LPS from *Escherichia coli*, PDG from *Staphylococcus aureus* and mucin type II from porcine stomach were moderately inhibitory at concentrations three orders of magnitude greater than for the most effective saccharides (0.10–0.49 mg/mL). Alginate, κ-carrageenans, fucoidan and citrus pectin were highly effective inhibitors characterized by the lowest IC50 (less than 0.002 mg/mL).

### 2.5. Bacterial Agglutination and Antimicrobial Activity

**Figure 4:**
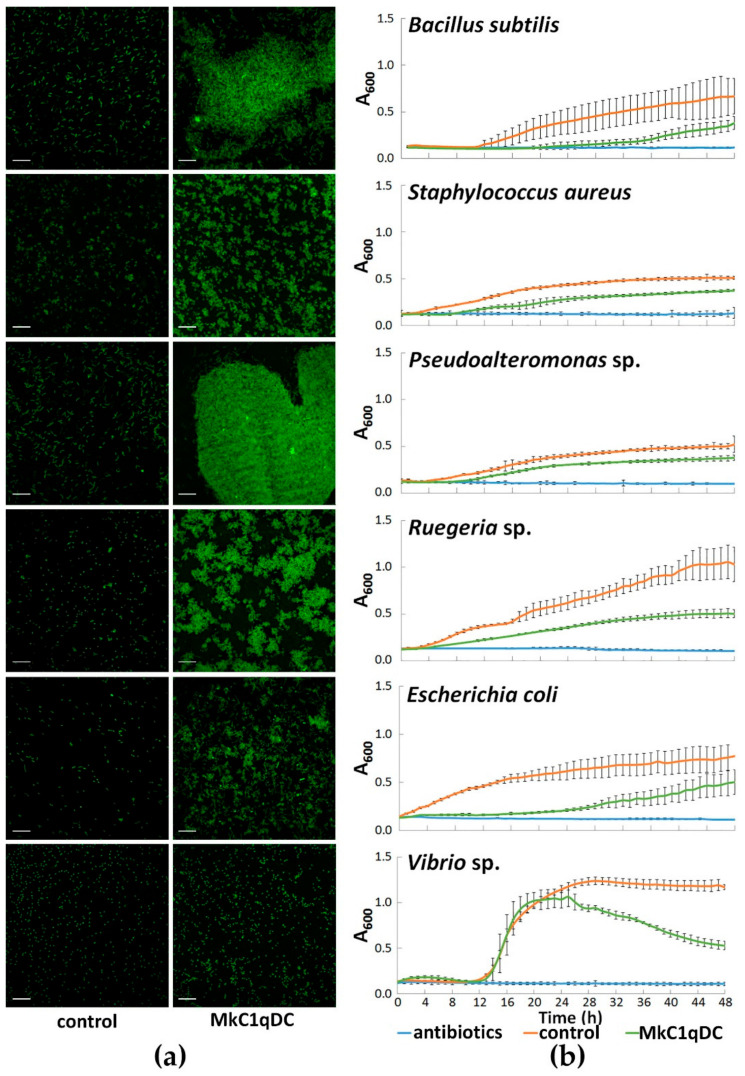
Antibacterial properties of MkC1qDC (0.1 mg/mL) against paraformaldehyde fixed FITC-labeled bacteria (**a**), and live bacterial cultures (**b**). The data on the graphs are presented as a mean ± 95% confidence interval. Scale bars: 10 µm.

MkC1qDC had antibacterial activity against both Gram-positive (*Bacillus subtilis*, *S. aureus*) and Gram-negative (*Vibrio* sp., *Ruegeria* sp., *E. coli*, *Pseudoalteromonas* sp.) bacteria (Figure 4). At the same time, agglutination occurred most effectively in the cases of *Pseudoalteromonas* sp. and *B. subtilis*, which in the presence of MkC1qDC formed the largest conglomerates, in contrast to *Vibrio* sp., which were united into small sparse groups of 4–15 cells. In addition, MkC1qDC showed bacteriostatic properties against most of the above strains as significant growth suppression started after 4–12 h (*p* < 0.05). The only exception was *Vibrio* sp. The decrease in the density of the *Vibrio* sp. cultures in the presence of MkC1qDC occurred only after 26 h of cultivation (*p* < 0.05) when the control was in a stationary phase (Figure 4).

### 2.6. Antibody Production and Immunohistochemical Localization of MkC1qDC in Mussel Tissues

**Figure 5:**
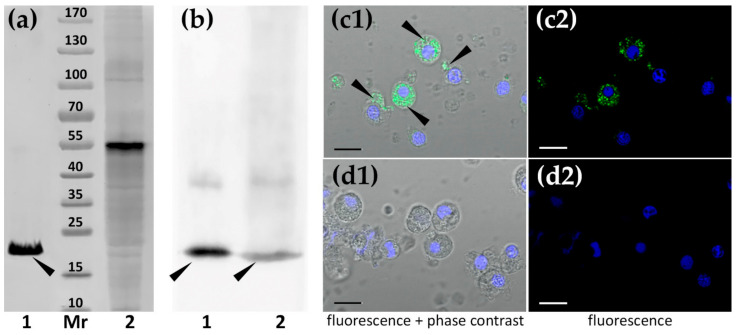
Detection of MkC1qDC (narrow labeled) in the components of M. kurilensis hemolymph: in plasma (**a**,**b**) and in hemocytes (**c**,**d**). (**a**) Electrophoresis; (**b**) Western blotting (1—purified MkC1qDC; 2—plasma; Mr—molecular weight standards); (**c1**,**c2**) treatment with MkC1qDC antibodies; (**d1**,**d2**) control with pre-immune rabbit serum. Nuclei are colored in blue by DAPI. Scale bars: 10 µm.

To further characterize MkC1qDC polyclonal antibodies were raised in rabbits. The antibody reacted strongly with purified MkC1qDC as well as a polypeptide with the same molecular weight in the plasma of *M. kurilensis* (Figure 5a,b). The upper ~40 kDa band appeared to be an incompletely dissociated dimer, as evidenced by its molecular weight, an increase in this fraction during storage and the disappearance of the component when 2-mercaptoethanol is replaced by dithiothreitol. Finally, antisera and purified immunoglobulin G fraction were tested in indirect enzyme-linked immunosorbent assay (ELISA) and Western blotting, and proved to be suitable for an investigation of tissue-specific MkC1qDC localization taking 1/500 dilution as a working titer.

The intracellular localization of MkC1qDC was most obvious in hemocytes (Figure 5c). A subset of the large cells had stained granules that could occupy almost the entire cytoplasm. In contrast, most of the agranulocytes and all small juvenile hemocytes were not stained. Control samples with pre-immune rabbit serum treatment showed no staining (Figure 5d).

**Figure 6:**
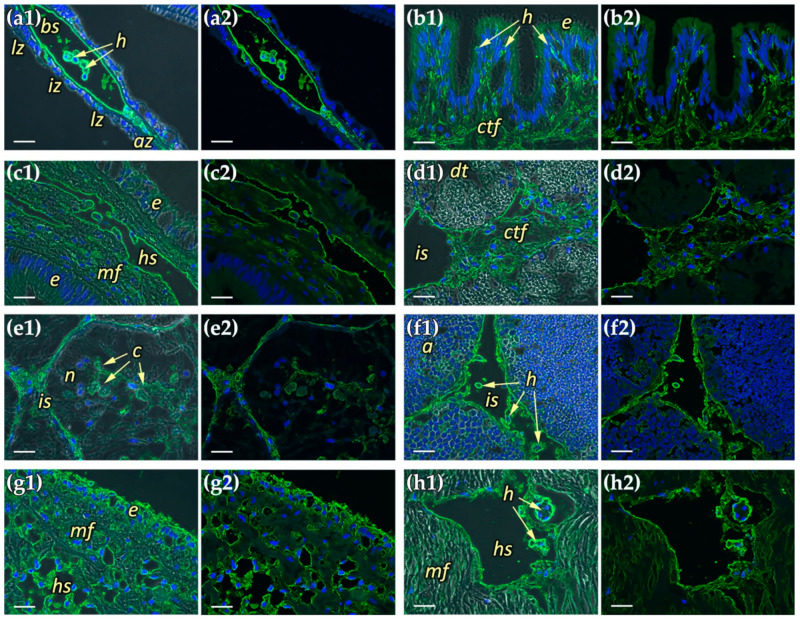
The MkC1qDC localization in organs of *M. kurilensis*: (**a1**,**a2**) gills; (**b1**,**b2**) mantle; (**c1**,**c2**) intestine; (**d1**,**d2**) digestive gland; (**e1**,**e2**) kidney; (**f1**,**f2**) gonad; (**g1**,**g2**) pericardium; (**h1**,**h2**) muscle; (**a1**–**h1**) fluorescence with phase contrast; (**a2**–**h2**) fluorescence only. Abfrontal zone: *az*; acinus: *a*; hemocytes: *h*; hemal sinuses/vessels: *hs*; intermediate zone: *iz*; bronchial sinus: *bs*; interstitial space: *is*; concretions: *c*; lateral zone: *lz*; muscle fibers: *mf*; nephrocytes: *n*; digestive tubule: *dt*; connective tissue fibers: *ctf*; epithelium: *e*. Scale bars: 20 µm.

In paraffin sections MkC1qDC was found in structures associated with the hemal system (Figure 6). The protein was mainly in hemocytes and the walls of hemal sinuses and vessels, as well as in the ECM of connective tissue and interstitial space. Gills were one of the organs expressing the highest levels of the protein. Intense immunofluorescent staining was well defined to the walls of bronchial sinuses inside the ctenidia. Moreover, hemocytes that were present in large numbers inside the sinus, including the abfrontal narrowing, were brightly stained and stood out, particularly compared to other organs (Figure 6a). Mantle, another organ with massive contact with the water environment, had some differences with respect to MkC1qDC localization. As in the previous case, staining was detected along the walls of the hemal sinuses and in the hemocytes located inside them. In addition, intensely stained hemocytes were found in the epithelial wall of the papillae. However, fibrous components were stained most evidently in the marginal zone of the mantle (Figure 6b). The distribution pattern of MkC1qDC in the internal epithelium of different parts of the intestine was identical: the walls of hemal sinuses and hemocytes located inside them or intercalated in between cells of the internal epithelium were stained most intensely (Figure 6c). The digestive gland of *M. kurilensis*, which is formed by the digestive tubules surrounded by the interstitium, hemocytes, as well as the walls of the interstitial space and fibers of relatively large fibrous formations, were positive for MkC1qDC (Figure 6d). The staining for MkC1qDC was weak in the kidney tubules, formed by nephrocytes with concretions and large vacuoles, while the relatively narrow interstitial space between the tubules, filled with fibrous tissue with hemocytes, was stained more intensely. In addition, the walls of concretions, which contain collagen, had slightly more intense staining compared to the control (Figure 6e). In gonads, which are mainly filled with gametes in different stages of development, intense fluorescence was confined to the interstitial space formed by the connective tissue, in which the walls and hemocytes were best stained (Figure 6f). In general, the intensity of the signal in the pericardium was intense, which might be explained by the abundance of hemal canals and hemocytes located within. Additionally, the pericardial epithelium was characterized by slightly more intense fluorescence (Figure 6g). Analysis of the posterior adductor muscle also showed the presence of MkC1qDC at the edge of the hemal vessels and in the hemocytes located inside them with a slight color of the tissue (Figure 6h).

### 2.7. Antiproliferative Activity on HeLa Cell Line

**Figure 7:**
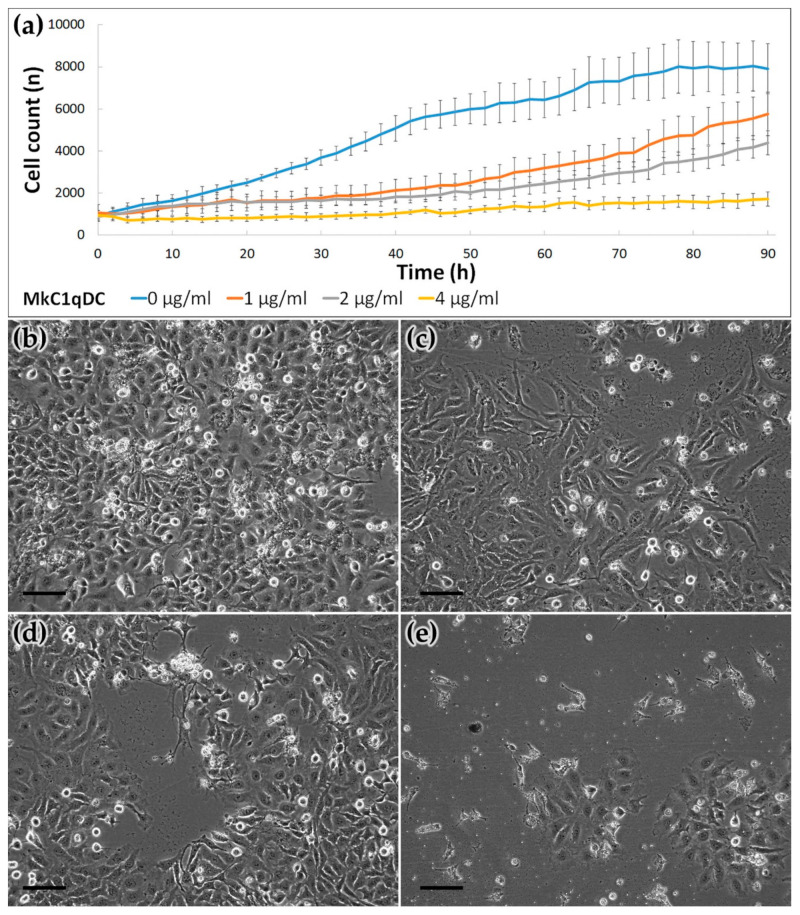
The MkC1qDC effect on HeLa cell line proliferation. Time-lapse cell monitoring and dynamic count based on Cell iQ machine learning during 90 h MkC1qDC treatment (**a**); the HeLa cell cultures after 90 h cultivation with MkC1qDC concentrations 0 μg/mL (control, **b**), 1 μg/mL (**c**), 2 μg/mL (**d**) and 4 μg/mL (**e**). Scale bars: 50 µm.

Finally, we tested if MkC1qDC may have antiproliferative activity in a mammalian context. To determine this, we monitored HeLa proliferation using high-content, time-lapse microscopy. We used a machine-learning algorithm to segment individual cells and determined cell proliferation in the presence or absence of MkC1qDC. Significant differences between control and treated groups were detected after 4 h (4 μg/mL of MkC1qDC) or 16 h (1 and 2 μg/mL) (Figure 7). Further analysis showed that MkC1qDCa elicited a dose-dependent antiproliferative effect. At the highest concentration, we detected next to no cell proliferation and most cells displayed morphology akin to dead cells.

## 3. Discussion

Using a HAI technique to study the carbohydrate specificity of the *M. kurilensis* plasma, we observed a strong inhibitory effect on D-glucuronic acids. Earlier, in a closely related species *Modiolus modiolus*, the same analysis did not reveal the same specificity [28]. Lectins modiolin H and E isolated from *M. modiolus* had a different molecular weight [28,29] from lectin-like MkC1qDC observed in this study (Figure 1b and Figure 3a). Obtaining N-terminal sequence of 41 amino acids, any significant homology matches were not identified, which indicates the uniqueness of the protein’s N-terminus. All obtained data indicate that a new protein with unique carbohydrate-recognizing properties has been isolated. From mass spectrometry and de novo assembling we observed the full-length MkC1qDC sequence with high similarity to MgC1q4 (Figure 2d–f). We find out that MkC1qDC has similar structure to the group of Bivalvia sialic acid-binding lectins determined as C1qDC [30]. Moreover, molecular weight and pI of MkC1qDC were very similar to that of other globular head C1qDC bivalve proteins isolated from *Chlamys farreri* (CfC1qDC) and *Argopecten irradians* (AiC1qDC-1) [8,9].

C1qDC proteins have been found to be immune factors in many cases. Various pathogens and their components induce an increase in C1qDC proteins expression, as well as C1qDC proteins demonstrating the ability to bind PDG, LPS, poly I:C, mannan, β-1,3-glucan, yeast glucan and live bacteria, generally indicating their role as PRRs [9,10,11,12,13,18,19,20,31,32,33,34,35,36,37,38]. Agglutinating activity of MkC1qDC was observed against Gram-positive and Gram-negative bacteria, but with slightly more pronounced selectivity for certain strains, such as *Pseudoalteromonas* sp. and *B. subtilis* (Figure 4). Agglutinating activity similar to that discussed in this study was observed for recombinant proteins from *C. farreri* (CfC1qDC) and *A. irradians* (AiC1qDC-1, AiC1qDC-2) scallops [8,9,11]. Interestingly, HA correlates with the content of the 19 kDa polypeptide in the *M. kurilensis* plasma [39], which probably means the importance of MkC1qDC as a soluble PRR with agglutinating function. 

It is currently known that C1qDC proteins in the bivalve can be expressed in in all organs [8,16,17,20,37], especially in hepatopancreas [9,11,19,33], hemocytes [12,13,40], mantle [12,18] and gills [19]. However, transcription in hemocytes invariably increased upon immune stimulation. This fact indicates the clearly inducible nature of C1qDC synthesis and their involvement in the immune response with hemolymph. MkC1qDC was found in all organs of the mollusk; however, the only structures with intracellular localization were hemocytes (Figure 5), which are ubiquitous in the elements of the hemal system or sometimes migrate in the epithelium of the mantle and the intestine. In all other cases, MkC1qDC was associated with extracellular structures, mainly with the walls of the hemal system and interstitial space, as well as with ECM fibers (Figure 6). Organs with the highest expression are the gills, the edge of the mantle and the pericardium—these are tissues with the highest exposure to pathogens. Thus, it can be assumed that the main cells synthesizing MkC1qDC are hemocytes, and due to good solubility, it circulates throughout the body and further can be fixed on the components of the extracellular matrix, performing immune or other possible additional functions.

The role of C1qDC proteins as PRRs implies their specificity for various PAMPs; this study also showed the high sensitivity of MkC1qDC towards mannan, LPS and PDG (Table 1). However, the specificity toward alginate, κ-carrageenan, fucoidan and pectin was significantly higher, making MkC1qDC protein characterized by most pronounced specificity to glycans enriched with acid galactans and mannans. These polysaccharides are mainly components of algal cell walls, which explains some of their structural similarity. Besides their obvious feeding role, microalgae can be invasive in bivalve and initiate some pathogenicity processes. In particular, earlier this was shown for *M. kurilensis* and *Coccomyxa parasitica* [41]. At the same time, for Chlorophyta the content of alginate and pectin in the cell walls is known [42]. Regarding selectivity to monosaccharides, the affinity of bivalve C1qDC proteins for sialic acid was only shown [17,18,30], while in the current study the highest activity was detected for sialic acid, but also for other acetylated monosaccharides such as uronic acids, as well as for D-galactose, 2-deoxy-D-galactose, L-gulose and disaccharides D-lactose and 2α-mannobiose. At the same time, many lectins showed the wide spectrum of carbohydrate specificity, especially for oligosaccharide with different structures [43,44]. It was also demonstrated that point amino acid substitutions in the carbohydrate-recognition domain of C-type lectin can lead to a significant change in affinity from mannose to methyl L-fucosides [45] or galactose [46] as well as a ricin-B chain-like galactose-binding protein which was obtained from sialic acid-binding lectin [47].

The range of carbohydrate specificity determines the potential in the use of lectins and lectin-like proteins in biotechnology and biomedical science. During tumor cells transformation, a change in the carbohydrates types of their surface occurs. Therefore, the lectins and lectin-like proteins that are able to recognize particular carbohydrate patterns also show antitumor activity. Impotence of cell surface sialylation in tumorigenesis and metastasis was known for a long time [48,49]. The sialic acid-binding lectin from the mollusk *Haliotis discus discus*, which is also a representative of the C1qDC proteins [30], possesses pronounced antitumor properties [50,51,52]. Other important saccharides for tumorigenic cell surface alteration are mannosides [53]. Hence, the mannose binding lectin was already used in designing a selective drug delivery system [54,55]. Additionally, oncodiagnostic applications of different lectins with affinity to β-branched galactosides, mannosides, as well as to sialylation and fucosylation glycoconjugates, was shown [56,57]. In the current study, MkC1qDC, which showed sialic acid binding lectin-like activity and the highest affinity to acidic galactans and mannans, significantly inhibited the growth of HeLa at the concentration 1 mg/mL, while at 4 mg/mL caused death of most of the cells, resulting in the absence of the significant growth of cell line even after 90 h of cultivation (Figure 7). This result is only a preliminary indicator of the biomedical potential of the new protein, and further analysis should include the study of both normal and cancer cell lines in order to identify the mechanisms of the antiproliferative activity of MkC1qDC.

Thus, this study first described a novel C1qDC protein, which has immune functions and is associated with the hemal system and interstitial compartment of the marine mollusk *M. kurilensis*. The PRR properties such as antibacterial activity and interactions with PAMPs (mannan, LPS, PDG) were observed. At the same time, agglutination activity and growth inhibition of bacteria had various effects on different strains, although MkC1qDC properties were not associated with their Gram classification. The investigated range of carbohydrate affinity of C1qDC protein has a unique character that, together with its ability to inhibit the growth of the HeLa cells, indicates possible implementation of MkC1qDC protein in biotechnology and biomedicine. Taking into account that the MkC1qDC protein is capable of recognizing acidic galactans and mannans, it may be of interest for creating systems for targeted delivery of therapeutic agents to cancer cells, and can also be included in diagnostic kits for phenotyping the cell surface carbohydrate profile. This is of interest for further research and development involving both native and recombinant MkC1qDC.

## 4. Materials and Methods

### 4.1. Purification and Electrophoretic Properties of the MkC1qDC

The MkC1qDC was purified from cell-free hemolymph (plasma) of *M. kurilensis*. Healthy adult mollusks with shells over 70 mm in length were selected from Peter the Great Bay, Sea of Japan (42.892078° N, 132.737502° E and 43.200788° N, 131.914084° E). Hemolymph was isolated immediately from the hemal sinus of the dorsal adductor muscle. Cells were removed by centrifugation for 15 min at 4 °C and 300× *g*. Plasma from different individuals was mixed, clarified by centrifugation for 30 min at 4000× *g* and 4 °C and stored at −80 °C until the next stage. 

The isolation of MkC1qDC was carried out in a few steps. Firstly, ammonium sulfate precipitation [58] of *M. kurilensis* plasma proteins with 0−15%, 15−30%, 30−45%, 45−60%, 60−75% and 75−85% salt saturation at 0 °C was produced. Protein coagulates were centrifuged for 30 min at 4000× *g* and 0 °C to precipitate. The obtained precipitates were dissolved in Tris buffer saline (TBS: 10 mM Tris-HCl, 150 mM NaCl, pH 7.5), dialyzed against a large volume of TBS three times at 4 °C, clarified by centrifugation (30 min, 10,000× *g*, 4 °C) and added CaCl_2_ up to 15 mM just before using. The target activity was determined by hemagglutination assays described below, and included inhibition by uronic (D-glucuronic, D-galacturonic) acids and adding chelants (EDTA, and EGTA) for Ca^2+^-dependence determination. 

Citrus pectin (Copenhagen Pectin A/S) covalently linked to Sepharose CL-4B (Sigma-Aldrich, St. Louis, MO, USA) by divinyl sulfone (Sigma-Aldrich, St. Louis, MO, USA) was applied for affinity chromatography on the final purification step. Loading of prepared fractions with target activity and column washing from unbound components was performed in TBS with 15 mM CaCl_2_. The elution was carried out with a large buffer solution containing a chelant (200 mM Tris-HCl, 150 mM NaCl, 30 mM Na_2_EDTA, pH 7.5). All stages were performed with the flow 0.2 mL/min. The eluate was dialyzed against TBS at 4 °C and divided into aliquots containing 100 μg of protein in cryovials, which were frozen and stored in liquid nitrogen at −196 °C.

Protein concentration was determined by the Bradford method [59], using BSA as standard, and measured by NanoPhotometer P360 (Implen) at a wavelength of 595 nm.

To analyze the quality of protein isolation at each stage, SDS-PAGE based on 12% polyacrylamide gel stained with Coomassie Brilliant Blue G-250 solution was produced [60].

The isoelectric point (pI) and composition of the purified MkC1qDC were determined by 2D electrophoresis using ReadyStrip™ IPG strips pH of 4−7 (Bio-Rad, Hercules, CA, USA) in the first dimension and a gradient of 4−20% polyacrylamide gel in the second dimension.

### 4.2. Amino Acid Sequencing

N-terminal sequencing was performed according to Edman’s degradation method [61] by a Procise 492 Protein Sequencer (Applied Biosystems, Waltham, MA, USA), using the manufacturer’s protocol. 

For mass spectrometry, MkC1qDC was dissolved in 6 M guanidinium-HCl, 100 mM Tris pH 8, 10 mM chloroacetamide, 5 mM Tris (2-carboxyethyl) phosphine hydrochloride, heated at 95 °C for 5 min. The sample was diluted with water 10-fold and 1/100 trypsin/substrate was added. Digestion was performed at 37 °C for 4 h. The digest was acidified to 1% trifluoroacetic acid final concentration, spun at 15,000× *g* for 10 min and desalted on homemade C18 stage tips as described previously [62]. Peptides were analyzed on a Fusion Lumos mass spectrometer (Thermo Fisher Scientific, Milan, Italy) coupled to an RSLnano uHPLC pump (Thermo Fisher Scientific, Milan, Italy). Peptides were separated on a 15 cm C18 Aurora column (IonOptiks, Victoria, Australia) using a 40 min gradient from 5−35% acetonitrile with 0.05% acetic acid. The instrument was operating in a high/high mode with a resolution of 120k/35k (MS and MS/MS). De novo sequencing was performed using the PEAKS software suite (Bioinformatics Solutions) with 5 ppm/0.01 Da mass accuracy (MS and MS/MS). Cysteine carbamylation fixed and Methionine oxidation (Variable) were selected as modification; trypsin was selected as protease with 2 missed cleavages. The list of peptides was trimmed using a cut-off of 80% Average Local Confidence Score. These high-confidence peptides were assembled into longer stretches using ALPS, a de Bruijn assembler, using a k-value of 7 as described [27]. This resulted in the assembly of 11 protein sequences. Initial BLAST searching against a non-redundant Bivalvia sequence database revealed a significant homology to CBX41653.1. Therefore, it was used as a template to stitch the peptides together by aligning them to CBX41653.1 using Clustal Omega [63]. The theoretical molecular weight and pI of assembled MkC1qDC were determined by ExPASy (http://web.expasy.org/compute_pi/, accessed on 26 May 2021).

### 4.3. Hemagglutination, Carbohydrate Specificity Assay, pH and Temperature Effects 

Hemagglutination (HA) was performed as described previously [64], using fixed human (0, A, B, AB), rabbit, rat and sheep erythrocytes. In the case of purified MkC1qDC the protein concentration of 0.1 mg/mL was used.

To investigate the carbohydrate specificity of MkC1qDC, a hemagglutination inhibition (HAI) test with the presence of one of 41 different saccharides was performed, 12 of which were polymers, 6 were oligomers and others were monomers (Table 1). Each carbohydrate was used in a series concentration of two-fold dilutions from 30 to 0.0029 mM (for polymers, concentration was calculated based on average monomers weight), and the inhibitory effect was determined by a decrease in the lectin HA activity (titer decline for one 2-fold dilution).

Isotonic (150 mM NaCl) buffer solutions were used to determine the pH dependence: glycine-HCl (pH 3), sodium acetate-acetic acid (pH 4, pH 5), sodium cacodylate-HCl (pH 6, pH 7), Tris-HCl (pH 8), glycine-NaOH (pH 9) and carbonate-bicarbonate (pH 10) at a concentration of 20 mM. HA activity was checked after 1 h of incubation at room temperature in TBS/Ca^2+^ with high buffering capacity (50 mM Tris-HCl, 150 mM NaCl, 15 mM CaCl_2_, pH 7.5).

To study the thermal lability, MkC1qDC samples were incubated for 1 h at temperatures of 0, 10, 20, 30, 40, 50, 60 and 70 °C and then were brought to room temperature before HA reaction.

### 4.4. Bacterial Agglutination and Bacteriostatic Assays

Gram-negative (*Vibrio* sp., *Ruegeria* sp., *E. coli*, *Pseudoalteromonas* sp.) and Gram-positive (*S. aureus*, *B. subtilis*) bacteria from marine organisms were used for analysis. Strains grew for 2-4 days to log-phase at room temperature in the liquid medium based on sterile-filtered sea water [65]. 

Agglutination assay was performed as described previously [66] with some modifications. After that, growing bacteria were collected by centrifugation (3000× *g*, 20 min at 4 °C), washed three times with HEPES buffer saline (HBS: 0.1 M HEPES-NaOH, 1.5 M NaCl, pH 7.4) and fixed by 4% PFA solution in HBS. Next, bacterial cells were stained with a fluorescent dye Fluorescein isothiocyanate (FITC, Thermo Fisher Scientific, Milan, Italy) with a final concentration of 0.1 mg/mL, vortexed at 800 rpm for 1 h, and washed 3 times in HBS. The stained suspensions with a final optical density (A_600_) of 1 were mixed in HBS with 50 mM CaCl_2_ and 0.1 mg/mL MkC1qDC, or without it as control (3 replicates for each condition). The mixtures were vortexed at 100 rpm for 1 h at room temperature. Visualization was performed using laser scanning microscope FV1200MPE-FV12M-5XX-3XX (Olympus). 

For the evaluation of MkC1qDC antimicrobial activity, bacteria were diluted in poor broth medium (PBM: 1.5% peptone, 1.5% NaCl, pH 7.2) to A_600_ = 0.05 and 50 μL placed into each well of the 96-well flat-bottom microplate. Then 50 μL of 0.2 mg/mL MkC1qDC solution in PBM was added in experimental wells, or 50 μL PBM as a positive growth control, or 50 μL antibiotic solution (penicillin 2000 units/mL and streptomycin 2 mg/mL) in PBM as negative control (6 replicates for each condition). The results of bacterial growth inhibition were estimated at 600 nm using the Cytation 5 Cell Imaging Multi-Mode Reader (BioTek). The inhibition efficiency was evaluated by the delay time in the bacteria growth in the presence of lectin vs the positive control.

### 4.5. Preparation and Validation of Polyclonal Antibodies 

The commonly used immunization protocols [67] with some modifications were employed to generate the rabbit polyclonal antibody to MkC1qDC. Briefly, the first intramuscular injection of purified MkC1qDC was performed with complete Freund’s adjuvant; then two consecutive injections of the immunogen with incomplete Freund’s adjuvant were given at intervals of 20 and 60 days, respectively; the boost was carried out in 14 days by a subcutaneous injection of MkC1qDC in sterile TBS. Each injection contained 250 µg of purified MkC1qDC. Blood was taken 10-14 days after each injection from the ear vein, and the isolated serum was stored at –80 °C.

The immunoglobulin fraction was purified by standard protocol [68] using ammonium sulfate precipitation and Sephadex G-25 (GE Healthcare, Chicago, IL, USA) gel filtration for desalting.

Primary antibody activity was measured by the commonly used protocol of indirect ELISA [69] with horseradish peroxidase-conjugated secondary antibody and o-phenylenediamine as substrate. Results were detected by iMark Microplate Absorbance Reader (Bio-Rad, Hercules, CA, USA) at 492 nm. 

In addition, antibody validation using Western blotting was performed by Mini Trans-Blot® Module for Mini-PROTEAN® Tetra Cell (Bio-Rad, Hercules, CA, USA). After electrophoresis, samples were transferred onto the PVDF membrane and placed for 2 h in a 3% BSA solution in TBS with 0.05% Tween-20 (TBST). The purified primary antibody was used at 1/2500, 1/5000 and 1/10000 dilutions (calculated based on ELISA result) in 0.5% BSA prepared on TBST, followed by incubation at 23 °C for 2 h. Then it was incubated with mouse antirabbit secondary antibody conjugated with horseradish peroxidase (Thermo Fisher Scientific, Milan, Italy) at 1/20000 dilution in TBST, and incubated for 1 h at 23 °C. After that, a substrate for chemiluminescent staining Pierce ECL Plus Western Blotting Substrate (Thermo Fisher Scientific, Milan, Italy) was applied to the membrane according to the industrial protocol. The result was recorded on a ChemiDoc Touch Imaging System (Bio-Rad, Hercules, CA, USA). Analysis was performed using native *M. kurilensis* plasma and purified MkC1qDC as samples.

### 4.6. Immunohistochemistry and Protein Localization 

Tissue fragments of the intestine, mantle, muscle, pericardium, gills, gonads, digestive gland and kidney were excised and immediately fixed for 2 h in paraformaldehyde (PFA) solution in phosphate buffered saline (PBS: 10 mM Na_2_HPO_4_-KH_2_PO_4_, 137 mM NaCl, 2.7 mM KCl, pH 7.4), washed 10 times in PBS and dehydrated through a progressive series of ethanol, infiltrated by xylene and embedded in paraffin. Tissue sections with a thickness of 10 μm were obtained using an HM-360 rotary microtome (MICROM International GmbH, Germany). The paraffin sections were dewaxed and washed 3 times in PBS with 0.05% Tween-20 (PBST) with further staining without drying.

Suspension of live hemocytes was obtained as previously described [64], diluted in RPMI-1640 culture medium (PanEco, Tokyo, Japan) and placed in an 8-well chamber (Ibidi GmbH, Gräfelfing, Germany) for 30 min at 10 °C for their adhesion. Then the cells were fixed for 10 min at 23 °C by adding a 16% PFA in artificial sea water (ASW: 460 mM NaCl, 9.4 mM KCl, 48.3 mM MgCl_2_, 6 mM NaHCO_3_, 10.8 mM CaCl_2_, 10 mM HEPES, pH 7.5) directly to the culture medium to a final concentration of 1.5%. Then the liquid was taken from the wells, 300 μL of cold methanol was added to them and left at 4 °C for 10 min, after which the chamber was left for longer storage at –20 °C. Immediately before staining, hemocyte preparations were washed 3 times with PBST solution.

Further, in order to permeabilize the membranes, PBS with 0.5% Triton X-100 was applied to the sections and hemocytes for 10 min at 23 °C. The primary antibodies in PBS with 0.5% BSA at a dilution of 1/500 were incubated with preparations for 2 h at 23 °C; secondary goat antibodies conjugated with Alexa Fluor-488 (Thermo Fisher Scientific, Milan, Italy) with the same dilution were incubated for 1 h at 23 °C; nuclei were stained by DAPI (4,6-diamino-2-phenylindole, dihydrochloride; Invitrogen) with a concentration of 1 μg/mL in PBS for 5 min at 23 °C. Between all steps, samples were washed 3 times for 10 min in PBST and, after the last step of staining, embedded in the water-soluble medium Mowiol 4-88 (Sigma-Aldrich, St. Louis, MO, USA). The described procedure with pre-immune rabbit serum as primary antibody was used as a control. The samples were analyzed using FluoView FV1200MPE-FV12M-5XX-3XX laser scanning microscope (Olympus).

### 4.7. Human Cell Culture and Proliferation Assay

Cell proliferation assay was performed using a high-content imaging system Cell-iQ MLF (CM Technologies). Human adenocarcinoma cell line HeLa (ATCC) was plated in tissue-culture grade 24-well plates at a density of 15,000 cells/cm^2^. The cells were cultured at 37 °C and 5% CO_2_ in Dulbecco’s Modified Eagle’s medium (DMEM, Gibco) supplemented with 10% FBS. The cells were cultured for their adhesion and growth stabilization for 18 h, then treated with 1, 2 and 4 μg/mL of MkC1qDC and monitored for 90 h applying time-lapse phase-contrast imaging. Untreated wells were utilized as a control sample group. All series were cultivated and analyzed in six replicates. Cell proliferation data are presented as growth curves indicating total cell count produced automatically based on machine learning. 

### 4.8. Experimental Design and Statistical Rationales

Proteomics experiments were conducted on one purified protein sample. For the analysis of MkC1qDC effect on bacteria and HeLa growing, the Mann–Whitney U test and Kruskal–Wallis H test were used. A *p*-value less than 0.05 was considered to be statistically significant. Experimental results are presented as a mean ±95% confidence interval.

## Figures and Tables

**Table 1:** Carbohydrates used in the hemagglutination inhibition with MkC1qDC.

Carbohydrates		IC50s
*Polysaccharides*	alginate	<0.30 μg/mL
	κ-carrageenan	<0.66 μg/mL
	fucoidan	<0.62 μg/mL
	pectin	1.6 μg/mL
	mannan	101 μg/mL
	LPS	125 μg/mL
	PDG	250 μg/mL
	mucin type II	493 μg/mL
	hyaluronic acid	―
	chitosan	―
	agarose	―
	dextran	―
*Oligosaccharides*	d-lactose	15 mM, 5.13 mg/mL
	2α- mannobiose	30 mM, 10.26 mg/mL
	n-acetyl-d-lactosamine	―
	d-melibiose	―
	d-maltose	―
	d-raffinose	―
*Monosaccharides*	N-acetylneuraminic (sialic) acid	3.75 mM, 1.16 mg/mL
	d-galacturonic acid	7.5 mM, 1.46 mg/mL
	d-glucuronic acid	7.5 mM, 1.46 mg/mL
	l-gulose	7.5 mM, 1.35 mg/mL
	2-deoxy-d-galactose	15 mM, 2.46 mg/mL
	d-galactose	30 mM, 5.40 mg/mL
	d-mannose	―
	d-glucose	―
	d-fucose	―
	l-fucose	―
	N-acetyl-d-galactosamine	―
	N-acetyl-d-glucosamine	―
	N-acetyl-d-mannosamine	―
	d-glucosamine	―
	α-methyl-d-glucopyranose	―
	l-rhamnose	―
	d-ribose	―
	myo-inositol	―
	dl-arabinose	―
	d-xylose	―
	l-sorbose	―
	methyl-β-xylopyranose	―
	d-glucurono-6,3-lactone	―

## Data Availability

The mass spectrometry proteomics data have been deposited to the ProteomeXchange Consortium with the dataset identifier PXD027507.

## Supplementary Materials

The following are available online at https://www.mdpi.com/article/10.3390/md19120668/s1, Figure S1: Mass spectrometry data of peptides using for alignment with N-terminus peptides obtained by Edman’s degradation (a, b) and for construction 70 amino acids sequence (c–e) with high homology to Bivalvia C1qDC proteins (see Figure 2), Figure S2: The full-length MkC1qDC amino acids sequence in fasta format.
