# Supplementary material for: A Novel C1q Domain-Containing Protein Isolated from the Mollusk Modiolus kurilensis Recognizing Glycans Enriched with Acidic Galactans and Mannans
Source: Mar Drugs. 2021 Nov 26;19(12):668. doi: 10.3390/md19120668 (PMC8706970; doi:10.3390/md19120668)
Supplement: Supplementary file 1 [file marinedrugs-19-00668-s001.zip › marinedrugs-1461086-supplementary.pdf]

Supplementary Information

A novel C1q domain-containing protein isolated from the mollusk *Modiolus kurilensis* recognizing glycans enriched with acidic galactans and mannans

Andrei V. Grinchenko[1], Alex von Kriegsheim[2], Nikita A. Shved[1,3], Anna E. Egorova[3], Diana V. Ilyaskina[3], Tatiana D. Karp[3], Nikolay V. Goncharov[1,3], Irina Yu. Petrova[1] and Vadim V. Kumeiko[1,3,*]

[1] A.V. Zhirmunsky National Scientific Center of Marine Biology, Far Eastern Branch, Russian Academy of Sciences, 690041 Vladivostok, Russia; grishagrin@mail.ru (A.V.G.), iupet@mail.ru (I.Yu.P.)
[2] The University of Edinburgh, EH4 2XU Edinburgh, United Kingdom; Alex.VonKriegsheim@ed.ac.uk
[3] Far Eastern Federal University, 690922 Vladivostok, Russia; nikitawayfarer@yandex.ru (N.A.Sh.), bioanna1995@gmail.com (A.E.E.), Ilyaskinadiana0506@gmail.com (D.V.I.), tachellabio@gmail.com (T.D.K.), goncharovnv.GN@gmail.com (N.V.G.), vkumeiko@yandex.ru (V.V.K.)
* Correspondence: vkumeiko@yandex.ru; Tel.: +7902-555-1821

Contents

Figure S1. Mass spectrometry data of peptides using for alignment with N-terminus peptides obtained by Edman's degradation (a, b) and for construction 70 amino acids sequence (c–e) with high homology to Bivalvia C1qDC proteins (see Figure 2).....................S2

Figure S2. The full-length MkC1qDC amino acids sequence in fasta format...S3

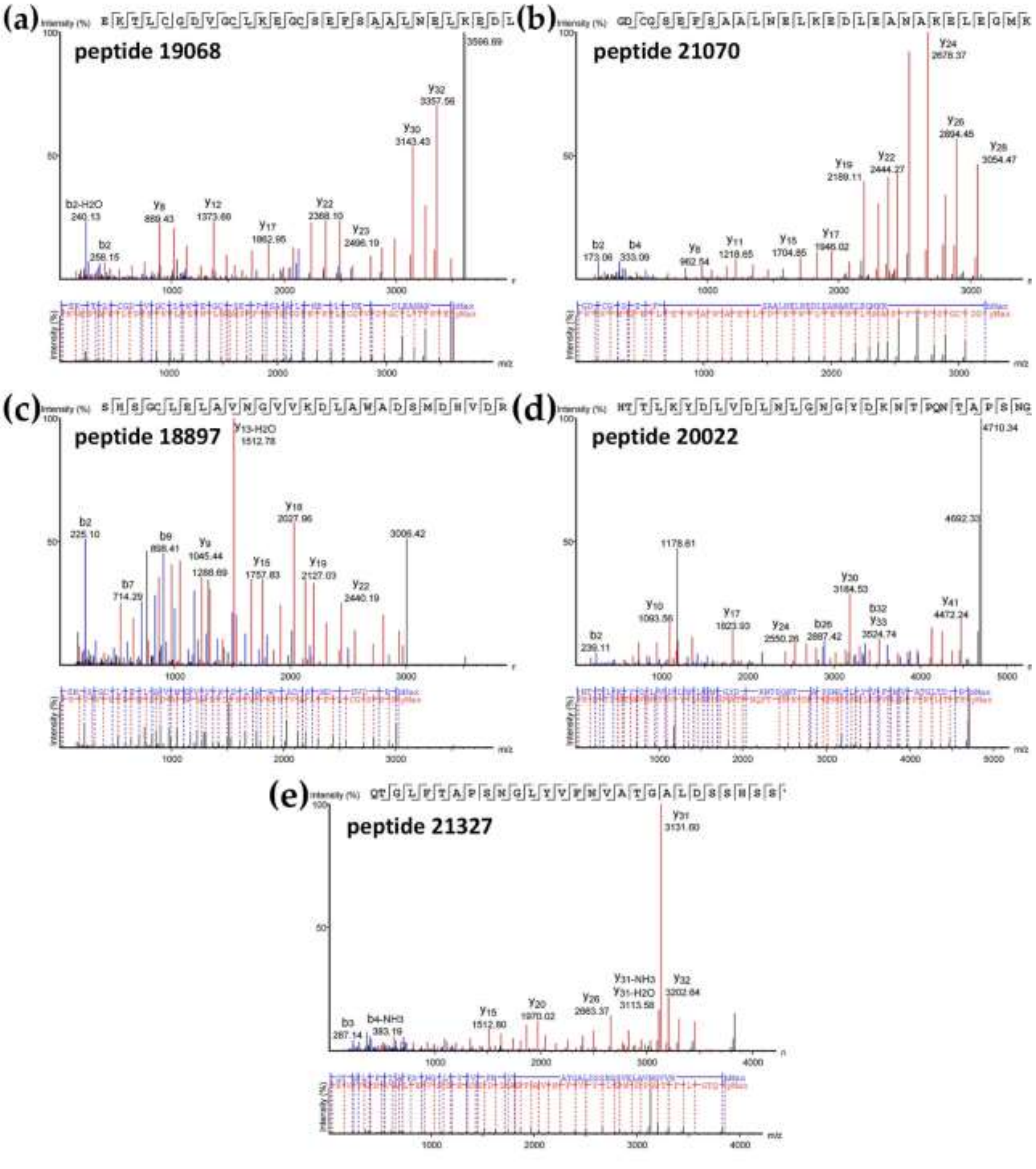

Figure S1. Mass spectrometry data of peptides used for alignment with N-terminus peptides obtained by Edman's degradation (a, b) and for construction of 70 amino-acids sequence (c–e) with high homology to Bivalvia C1qDC proteins (see Figure 2).

```
>MkC1qDC_Modiolus_kurilensis

EKTLCDVCLKECSEFSAALNELKEDLETNAKELAGMKKNNNAVAFYAYLSKSLPLNSVSKHTTLKYDLVD

LNLGNGYDKQTGLFTAPSNGLYVFNVATGAQDSSHSCLELAVNGVVKDLTWADSMDHVDRAFATTATPMS

LNENDKVLARLGEAHGGNELESNKYLRTSFSGFKVQ
```

Figure S2. The full-length MkC1qDC amino acids sequence in fasta format.
